# Reduced circulating anti-CXCR3 antibodies as a common hallmark bridging systemic autoimmunity and atherosclerosis

**DOI:** 10.3389/fimmu.2026.1937388

**Published:** 2026-09-03

**Authors:** Daniel Miranda-Prieto, Mercedes Alperi-López, Ángel I. Pérez-Álvarez, Silvia Suárez-Díaz, Sara Alonso-Castro, Harald Heidecke, Ana Suárez, Gabriela Riemekasten, Javier Rodríguez-Carrio

**Affiliations:** 1 Area of Immunology, Department of Functional Biology, Faculty of Medicine, University of Oviedo, Oviedo, Spain; 2 Basic and Translational Research on Inflammatory Diseases, Department of Metabolism, Instituto de Investigación Sanitaria del Principado de Asturias (ISPA), Oviedo, Spain; 3 Department of Rheumatology, Hospital Universitario Central de Asturias, Oviedo, Spain; 4 Department of Neurology, Hospital Universitario Central de Asturias, Oviedo, Spain; 5 Department of Internal Medicine, Hospital Universitario Central de Asturias, Oviedo, Spain; 6 CellTrend GmbH, Luckenwalde, Germany; 7 Department of Rheumatology and Clinical Immunology, University Medical Center Schleswig-Holstein Campus Lübeck, Lübeck, Germany

**Keywords:** arthritis (including rheumatoid arthritis), atherosclerosis, autoantibodies, autoantibodies-blood, biomarker, Sjogren, Sjogren’s disease

## Abstract

**Introduction:**

immune dysregulation underlies cardiovascular risk excess in systemic autoimmune diseases, such as rheumatoid arthritis (RA) and Sjögren disease (SjD). However, exact mediators are unknown. Regulatory autoantibodies targeting G protein–coupled receptors, including CXCR3, have emerged as modulators of immune and vascular homeostasis, but their role in autoimmunity remains ill-defined. Our aim was to evaluate anti-CXCR3 levels in systemic autoimmunity and their potential value as biomarkers.

**Methods:**

anti-CXCR3 IgG serum levels were quantified in early RA (n=84), clinically-suspect arthralgia (n=14), and controls (n=65). Established RA (n=103) and SjD (n=44) were recruited for validation. Atherosclerosis was assessed by carotid ultrasound. Cytokines were measured by multiplex immunoassays. Cardiometabolic-related proteins were evaluated using high-throughput targeted proteomics. Publicly available datasets were used for validation.

**Results:**

anti-CXCR3 antibodies were significantly reduced in early RA and clinically-suspect arthralgia compared with controls, independently of disease activity, autoantibodies, or systemic inflammation. This finding was confirmed in the validation cohort. Equivalent results were also observed in SjD. Anti-CXCR3 were negatively associated with good therapeutic outcomes upon csDMARD at 6 and 12 months in early RA. Lower anti-CXCR3 levels were independently associated with atherosclerosis occurrence and extent across conditions. Incorporating anti-CXCR3 into mSCORE improved risk stratification. Anti-CXCR3 were related to proteomic signatures linked to immune activation and to apoptosis, chemotaxis, and cell adhesion in an atherosclerosis-dependent manner in RA and SjD. Transcriptomic analyses indicated compartment-specific CXCR3 dysregulation both in RA and SjD.

**Conclusion:**

reduced anti-CXCR3 antibodies represent a shared hallmark bridging systemic autoimmunity and atherosclerosis burden, shaping our understanding on the regulatory role of antibodies at the vascular–immune interface. Clinical translation of anti-CXCR3 antibodies hold promise to improve risk stratification.

## 1 Introduction

Autoimmune diseases, such as rheumatoid arthritis (RA) or Sjögren disease (SjD) are hallmarked by a substantial cardiovascular disease (CVD) risk excess (1). Chronic immune dysregulation and autoimmune phenomena underlie risk excess, and accumulating evidence points to shared mechanisms among conditions (1, 2). However, precise immunopathogenic pathways linking systemic autoimmunity and atherosclerosis remain only partially understood.

Humoral immune responses have been proposed as central contributors to CVD (3). Increased levels of autoantibodies found in atherosclerosis mice model suggests an active role as drivers of atherosclerosis, but antigen-antibody axes are ill-defined (1, 4), and B-cell subset modulation led to opposed, even contradictory results, hence adding another layer of complexity of antibody-mediated regulation in vascular disease.

Autoantibodies against G-coupled protein receptors (GPCRs) have recently emerged as central players of immune and tissue homeostasis and key contributors to different conditions such as autoimmune diseases, infections, and neuroimmune disorders (5). These autoantibodies can modulate GPCRs signaling through activation, inhibition or biased activation of GPCRs or their ligands (6), thereby fine-tuning receptor activity and downstream cellular responses. However, although mechanistic studies are available, how they inform clinically relevant circuits remain unexplored. Integrative studies using omic approaches may help capturing functional relevance of these antibodies.

GPCRs are involved in a broad range of biological processes related to atherosclerosis. Autoantibodies against GPCRs such as angiotensin II type-1 receptor, involved in vascular damage and systemic inflammation, and against β1AR, a GPCR expressed in cardiac and immune cells, have been associated with CVD [reviewed in (5, 7)]. More recently, antibodies targeting CXCR3 have been shown to predict CV risk (8), likely through skewed Th1 activation. Although already linked to lung manifestations in systemic sclerosis (SSc) (9), evidence on the role of anti-CXCR3 in systemic autoimmune diseases is rather limited, especially in RA. Moreover, whether altered levels of these antibodies can be found in the earliest stages of the disease remains unexplored.

Given the unmet need for biomarkers that integrate systemic immune dysregulation and atherosclerosis in systemic autoimmunity, anti-CXCR3 antibodies represent a promising candidate. Although mechanistic evidence has been demonstrated in preclinical studies, limited evidence on their clinical relevance as disease markers is available. Therefore, the main aims of the present study were to assess anti-CXCR3 antibody levels in RA and SjD patients, to evaluate potential associations with atherosclerosis burden, and to investigate their role as a biomarker for CV risk stratification.

## 2 Materials and methods

### 2.1 Study participants

Early RA patients fulfilling 2010 ACR/EULAR classification criteria (10) or clinically suspect arthralgia (CSA) (11) were recruited at disease diagnosis. Early RA patients were not exposed to any disease-modifying antirheumatic drugs (DMARDs) at recruitment, and disease duration can be considered zero due to recruitment at diagnosis. A separate cohort of established RA patients (disease duration >1 year) was included as validation cohort (12). Additionally, a group of 13 biological-naïve RA patients was prospectively followed at baseline and 3 months after anti-TNF therapy. A cohort of SjD patients (13) was recruited. Healthy controls (HC) were recruited from the same population (see Supplementary Materials for detailed information on patient recruitment and procedures).

The study was approved by the institutional review board (Comité de Ética de Investigación con Medicamentos del Principado de Asturias) in compliance with the Declaration of Helsinki (reference CEImPA 2021.126).

### 2.2 Echocardiographic study

Atherosclerosis burden (plaque presence and number) was assessed by Doppler ultrasound as previously described (14) (Supplementary Material).

### 2.3 Anti-CXCR3 assessment

Levels of anti-CXCR3 IgG antibodies were quantified in serum samples using a commercial assay (CellTrend), following the instructions provided by the manufacturer (Supplementary Material).

### 2.4 Proteomic platform

Serum proteomics were carried out using a high-throughput analysis by means of the Proximity Extension Assay (15) (**Supplementary Material**).

### 2.5 Statistical analysis

Variables were summarized as median (interquartile range) or mean ± standard deviation, or *n*(%). Differences among groups were evaluated by Mann-Whitney *U*, Kruskal–Wallis, or Wilcoxon paired tests, according to the distribution of the variables.

The associations between anti-CXCR3 levels and atherosclerosis were analyzed by univariate and multivariate logistic regression adjusted by confounders (namely, traditional CV risk factors [sex, age, hypertension, diabetes, smoking and dyslipidemia], and treatment exposure and disease duration, when applicable (established disease). Detailed information on biomarker analysis, performance on risk stratification, proteomic analyses and gene expression datasets is included as **Supplementary Material**.

A p-value <0.050 was considered as statistically significant. Statistical analyses were carried out under SPSS v. 27, R v.4.1.3 and GraphPad Prism 8.0.

## 3 Results

### 3.1 Anti-CXCR3 antibodies in patients with systemic autoimmune diseases

**Figure 1:**
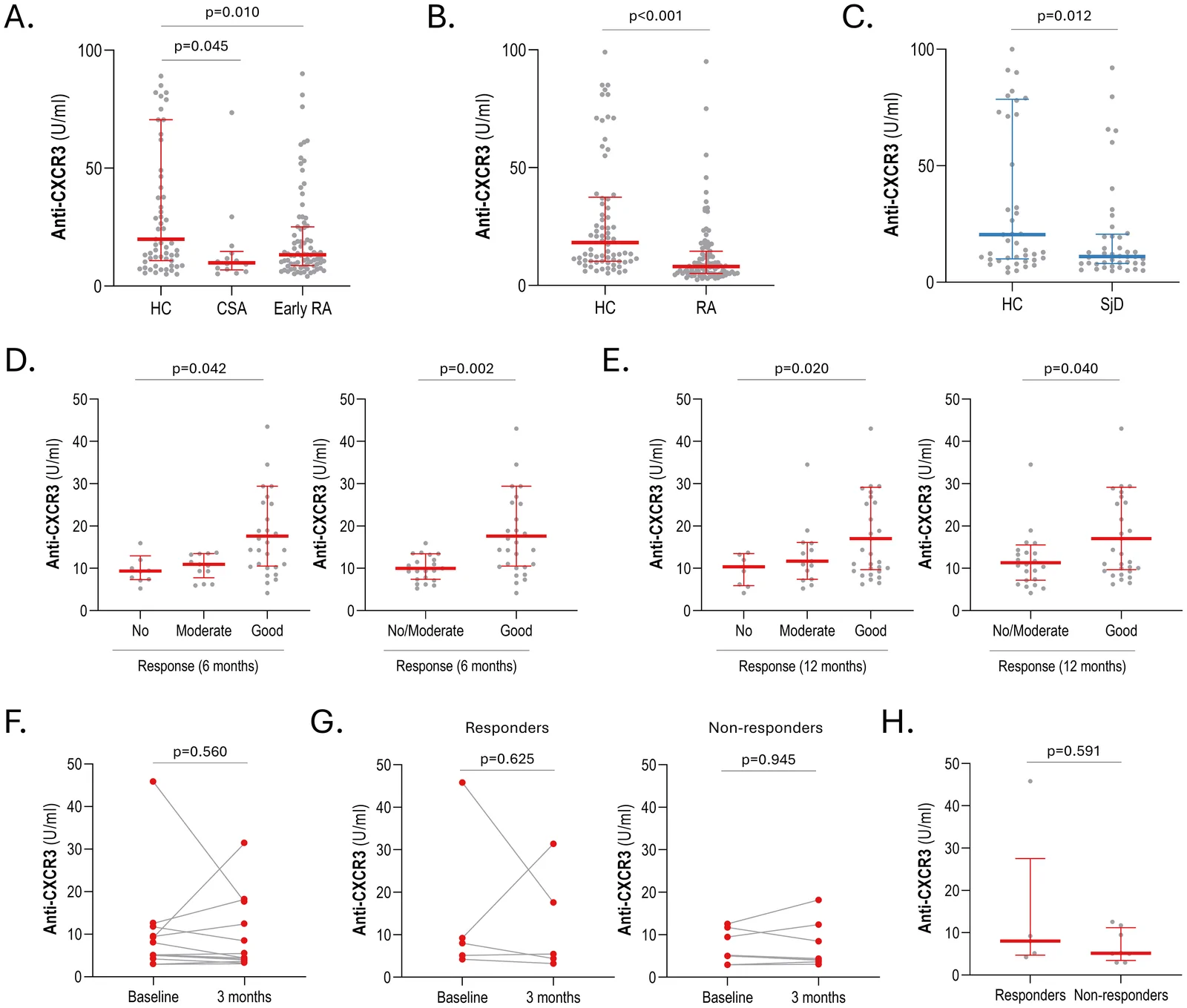
Anti-CXCR3 antibodies in systemic autoimmune diseases. The serum levels of anti-CXCR3 antibodies were compared in early RA (n=82), CSA (n=14) and HC (n=65) **(A)**, established RA (n=103) and HC (n=80) **(B)**, SjD patients (n=44) and HC (n=45) **(C)**. The serum levels of anti-CXCR3 antibodies were compared in early RA patients according to clinical outcomes (EULAR criteria) at 6 **(D)** or 12 months **(E)** upon csDMARD inhibition. Anti-CXCR3 antibodies were measured at baseline and 3-months after TNF blockade initiation in biological-naïve RA patients (n=13) **(F)**, and according to clinical outcomes **(G)**. Differences at baseline were also assessed **(H)**. In scatter plots, each dot represents one individual. Bars represent 25th percentile (lower), median and 75th percentile (upper). Color code depicts RA (red) or SjD (blue) data. Differences were assessed by Kruskal-Wallis (with Dunn-Bonferroni correction for multiple comparisons), Mann-Whitney U tests, or paired Wilcoxon paired test, as appropriate. P-values were indicated.

A total of 82 early RA patients, 14 CSA individuals and 65 HC were recruited for this study (**Supplementary Table 1**). The levels of anti-CXCR3 antibodies were found to be decreased in early RA and CSA groups compared to HC (**Figure 1A**). This result was confirmed in the validation cohort (**Figure 1B**), composed by patients with established RA (**Supplementary Table 2**). Furthermore, equivalent findings were observed in the analysis of the SjD cohort (**Figure 1C**; **Supplementary Table 3**).

Anti-CXCR3 antibodies were found to be unrelated to demographic and clinical features in early RA patients, including disease activity and symptom duration (**Table 1**). Disease-related autoantibodies (RF and ACPA) failed to show an association with anti-CXCR3 antibodies, whereas ANA positivity was associated with lower anti-CXCR3 levels (**Table 1**). No associations with the total level of immunoglobulins or complement levels were found (**Table 1**). Finally, anti-CXCR3 serum levels failed to show an association with proinflammatory cytokines, including B-cell related factors, in early RA (**Supplementary Table 4**).

**Table 1:** Anti-CXCR3 antibodies and clinical features in early RA.

Parameter	Association with anti-CXCR3"
Age	r=-0.173 p=0.120
Gender	p=0.433
Clinical features	
Duration of symptoms	r=-0.003 p=0.980
Morning stiffness	r=-0.067 p=0.549
Tender joint count	r=-0.044 p=0.697
Swollen joint count	r=-0.044 p=0.694
Patient global assessment	r=-0.215 p=0.054
Pain	r=-0.124 p=0.270
DAS28	r=-0.061 p=0.589
HAQ	r=-0.195 p=0.083
RF	p=0.948
ACPA	p=0.999
ANA	**p=0.** **037**
Laboratory parameters	
ESR	r=-0.063 p=0.576
CRP	r=-0.106 p=0.345
Total cholesterol	r=-0.105 p=0.349
HDL-cholesterol	r=-0.142 p=0.204
LDL-cholesterol	r=-0.050 p=0.653
Triglycerides	r=-0.076 p=0.505
IgG	r=0.169 p=0.192
IgA	r=0.190 p=0.142
IgM	r=-0.154 p=0.236
C3	r=-0.034 p=0.775
C4	r=-0.083 p=0.481

Associations between serum levels of anti-CXCR3 antibodies and demographic, clinical features, and laboratory parameters were analysed by Spearman’s rank tests or Mann-Whitney U tests in early RA patients. Coefficients (r) and p-values, or p-values for the difference between groups are shown. Those reaching statistical significance were highlighted in bold.

Lower levels of anti-CXCR3 antibodies at baseline were observed in early RA patients experiencing poor therapeutic response at 6 months upon csDMARD initiation (n=28) compared to those exhibiting good responses (n=30) (**Figure 1D**). Equivalent results were observed at 12 months (**Figure 1E**). On the contrary, anti-CXCR3 levels were unchanged at 3 months upon TNFi initiation (**Figure 1F**) (**Supplementary Table 5**), irrespectively of treatment outcomes (**Figures 1G, H**). These data should however be interpreted with caution, as exploratory and hypothesis-generating due to short follow-up and limited power to detect modest changes.

Taken together, these findings suggest that reduced anti-CXCR3 levels may be a common hallmark across systemic autoimmune conditions. Decreased anti-CXCR3 antibodies were found along the whole RA continuum, including the earliest stages, and connected with therapeutic outcomes.

### 3.2 Anti-CXCR3 antibodies and atherosclerosis burden in autoimmunity

Next, whether circulating anti-CXCR3 levels were associated with atherosclerosis burden and risk factors was investigated.

**Figure 2:**
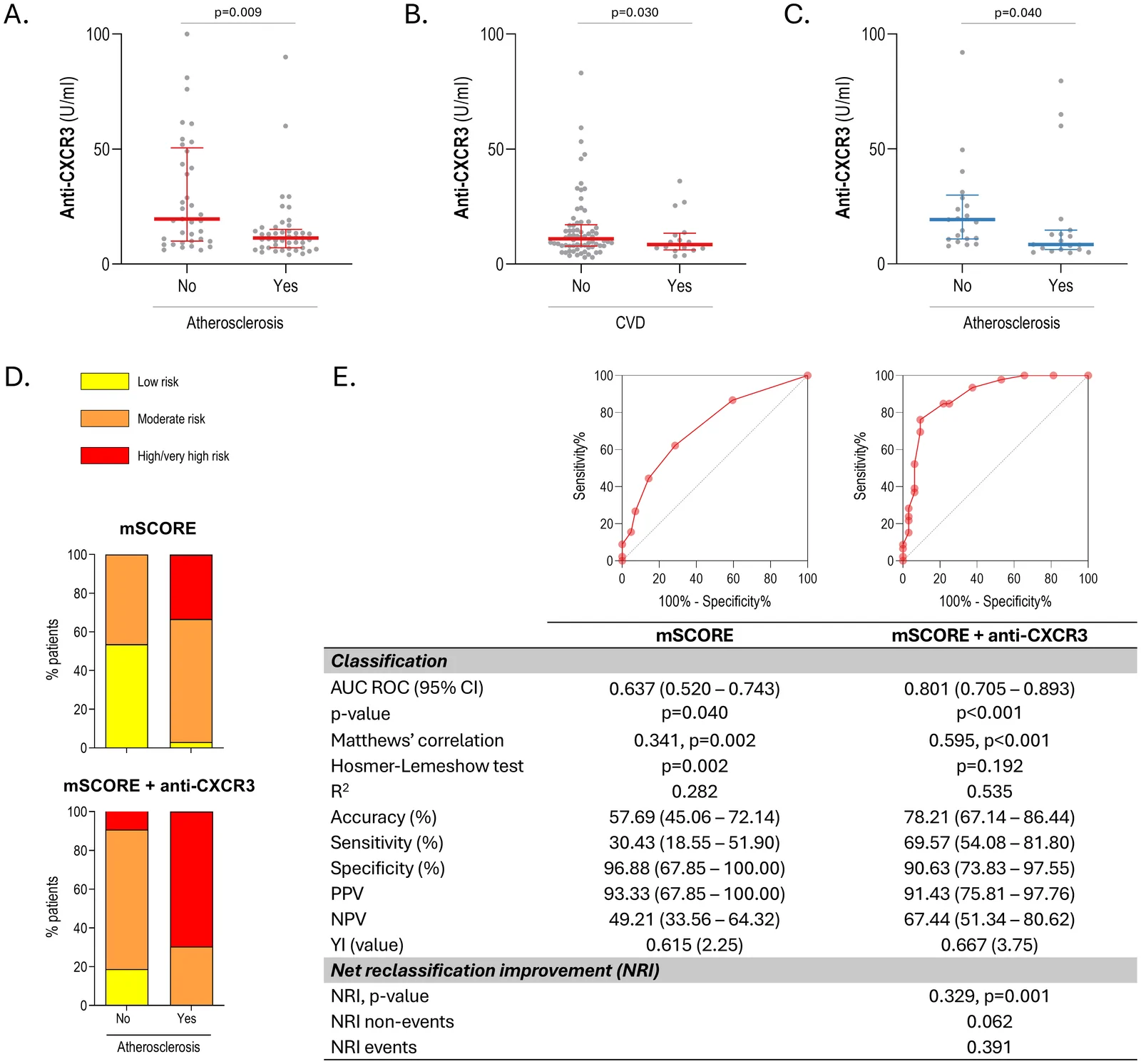
Anti-CXCR3 and atherosclerosis burden in systemic autoimmune diseases. The serum levels of anti-CXCR3 antibodies were compared according to atherosclerosis status in early RA patients **(A)**, according to CVD status in established RA **(B)**, and atherosclerosis status in SjD **(C)**. Bars represent 25th percentile (lower), median and 75th percentile (upper). Color code depicts RA (red) or SjD (blue) data. Differences were assessed by Mann-Whitney U tests. P-values were indicated. **(D)** Adding anti-CXCR3 strata to existing instrument (mSCORE + anti-CXCR3) improved risk stratification in early RA into more realistic risk groups, particularly in patients with atherosclerosis, compared to original tool (mSCORE), based on risk category distribution (low, moderate, and high/very high) and atherosclerosis status. **(E)** Classification, diagnostic metrics and reclassification measures (NRI) were calculated to evaluate incremental value. ROC curves for each model are shown at the top of the table.

Anti-CXCR3 levels did not exhibit associations with traditional CV risk factors (hypertension: p=0.085, dyslipidemia: p=0.345, diabetes: p=0.876, smoking: p=0.075, and obesity: p=0.789) in early RA. Patients with atherosclerosis exhibited lower anti-CXCR3 antibodies than their atherosclerosis-free counterparts (**Figure 2A**). Patients with high-risk plaques according to their echocardiographic characteristics exhibited lower anti-CXCR3 levels than those presenting with low-risk plaques (15.19 (13.28) vs 10.24 (7.09) U/ml, p=0.002). Furthermore, anti-CXCR3 levels were negatively associated with plaque number (r=0.248, p=0.020), although no associations were found with cIMT (r=0.139, p=322). The analysis of CVD history in the validation cohort confirmed these findings (**Figure 2B**). Regression analyses demonstrated that anti-CXCR3 levels were independent predictors of atherosclerosis occurrence in univariate or multivariate models in early RA, after adjusting for traditional CV risk factors (**Table 2**). Adjustment for statin therapy (n=17) did not change these findings [anti-CXCR3, OR (95% CI): 0.940 (0.885, 0.992), p=0.020]. Equivalent results were obtained when plaque number was modeled in fully-adjusted multivariate negative binomial regression analyses [b (95% CI): -0.026 (-0.032, -0.010), p=0.045], thereby confirming the independent association between anti-CXCR3 levels and atherosclerosis presence and extent in early RA. These findings were also confirmed in the validation cohort (**Supplementary Table 6**), even after adjusting for treatment exposure (**Supplementary Table 7**). Moreover, a similar picture was also obtained in SjD (**Figure 2C**) (**Supplementary Table 8**). Adjusting for treatment exposure did not change these findings (**Supplementary Table 9**). Finally, further adjustment for CRP or ESR levels did not significantly influence these associations (data not shown).

**Table 2:** Anti-CXCR3 antibodies as predictors of atherosclerosis in RA.

Model	OR	95% CI	P-value
Univariate			
Anti-CXCR3, per unit	0.967	0.942 – 0.993	**0.012**
Multivariate			
Anti-CXCR3, per unit	0.939	0.890 – 0.990	**0.019**
Sex, women	0.219	0.034 – 1.412	0.111
Age, per year	1.081	1.010 – 1.157	**0.025**
Smoking, yes	3.728	0.924 – 15.030	0.064
Hypertension, yes	1.751	0.395 – 7.778	0.461
Diabetes, yes	0.001	0.000 – 0.001	0.998
Dyslipidemia, yes	1.395	0.924 – 5.973	0.654

The role of anti-CXCR3 levels as predictor of atherosclerosis occurrence in early RA patients was analysed by univariate and multivariate logistic regression models. Odds ratio (OR), 95% confidence intervals (95% CI) and p-values for each predictor were shown. Those reaching statistical significance were highlighted in bold.

**Figure 3:**
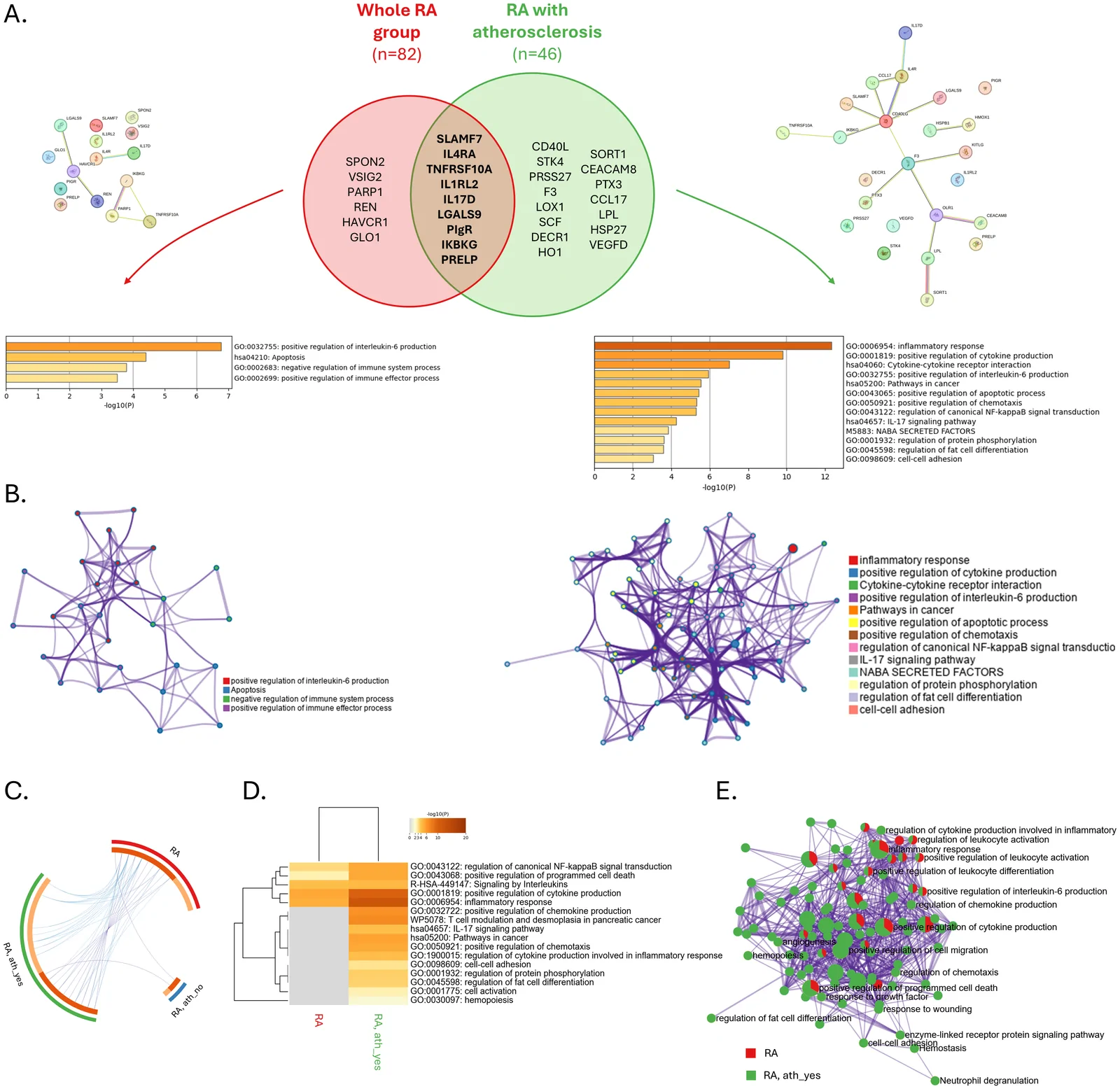
Proteomic analyses of anti-CXCR3 autoantibodies in RA and atherosclerosis status. Protein hits found to be associated with anti-CXCR3 antibody serum levels in the whole RA group (n=82) and in the subgroup of RA patients with atherosclerosis (n=46) were analyzed. Overlap analysis was shown as a Venn diagram **(A)**. Shared and group-specific proteins are listed in the overlap and non-overlap regions, respectively. Protein-protein interaction networks for the corresponding protein sets were generated using STRING. Pathway and process enrichment analysis was conducted under Metascape using KEGG pathway, GO biological processes, Reactome Gene Sets, Canonical Pathways, CORUM, and PANTHER Pathway as ontology sources. P-values for each term was calculated based on the accumulative hypergeometric distribution. To further capture the relationships among terms, enriched terms were selected and rendered as network plots **(B)**, where terms with similarity>0.3 were connected by edges. The networks were visualized using Cytoscape, where each node represented an enriched term and it is depicted by its cluster ID (see color legend). For group-wise analyses, circus plots **(C)** were used to shown to visualize and compare protein overlaps among the whole RA group, and patient subgroups according to atherosclerosis status (outside arcs). Inner arcs depicted protein usage: shared (dark orange) and unique (light orange). Purple lines link proteins shared by multiple groups. The greater the number of purple links and the longer the dark orange arcs implies greater overlap among groups. Blue lines link proteins that, although different, fall under the same ontology term across groups (that is, functional overlap among protein sets). To compare pathway and process enrichment patterns among groups, a heatmap of enriched terms with hierarchical clustering (kappa threshold: 0.3) was produced **(D)**. The heatmap cells were colored by their p-values. Integrated network layout of enriched terms across groups was derived to highlight shared and group-specific functional modules **(E)**. Each term is represented by a node, where size is proportional to the number of protein sets that fall under that term, and color code represents a pie chart proportional to number of hits from the protein sets. Main terms were labeled. Color code depicts whole RA group (red) or RA patients with atherosclerosis (green) and RA patients without atherosclerosis (green) data. RA, whole RA group; ath_yes, RA patients with atherosclerosis; ath_no, RA patients without atherosclerosis.

Our analyses revealed that anti-CXCR3 alone were able to discriminate between early RA patients with and without atherosclerosis [AUC ROC (95% CI): 0.671 (0.545-0.797), p=0.009]. We therefore evaluated if anti-CXCR3 antibodies could be useful in risk stratification. Adding anti-CXCR3 tertiles to the mSCORE (mSCORE + anti-CXCR3), improved risk stratification into more realistic risk categories (**Figure 2D**). The analysis of diagnostic and classification statistics supported this observation (**Figure 3B**). A better discrimination capacity [difference between areas = 0.164 (0.008–0.247), p<0.001], and improved classification metrics (accuracy, sensitivity, and Matthews’ Correlation coefficient) (**Figure 2E**) was achieved. NRI analysis confirmed a better reclassification performance, with a noticeable effect for those presenting atherosclerosis and a marginal to no effect in the atherosclerosis-free group (**Figure 2E**).

All these findings reinforce an independent association between decreased anti-CXCR3 antibodies and atherosclerosis in autoimmune conditions, also exhibiting an incremental value for risk stratification over existing instruments.

### 3.3 Anti-CXCR3 antibodies and proteomic signatures

Next, in order to get a more functional insight into the mechanisms behind anti-CXCR3 alterations, the associations between anti-CXCR3 levels and serum proteomic signatures were evaluated.

The levels of anti-CXCR3 antibodies were correlated with a number of protein hits in early RA (n=15). These proteins exhibited a significant protein-protein interaction (p=0.005, local average clustering coefficient=0.467) (**Figures 3A**, left). Pathway process enrichment analysis using Metascape uncovered a total of four functional pathways participated by these proteins, overall related to immune activation processes (**Figure 3A**). Subgroup analyses according to atherosclerosis status revealed a larger set of proteomic correlates (n=24) in patients with atherosclerosis, which were also strongly interconnected (PPI: p=1.25·10^-5^, local average clustering coefficient=0.475) (**Figures 3A**, right). Proteomic correlates in the atherosclerosis-free group were negligible (n=3). Protein traits differentially associated with the atherosclerosis group included species functionally related with CVD (LOX-1, F3, HO-1, SPRT1, CEACAM8, PTX3, LPL, HSP27 and VEGFD), in addition to those related to broad immunological processes. Pathway analyses restricted to this patient subset identified a number of events intimately linked to atherosclerosis, such as apoptosis, chemotaxis, fat cell differentiation and cell adhesion, as well as processes related to intracellular signaling pathways (**Figures 3A**, right).

Network analyses of terms retrieved in pathway and process enrichment analysis highlight clear differences in the structure and coherence of the biological signals (**Figure 3B**). The network derived from the whole RA group showed fewer connections and more dispersed functional terms, suggesting a broader and less coordinated set of processes. In contrast, the network derived from the group with atherosclerosis displayed a larger number of enriched processes with stronger interconnectivity, forming tighter clusters of related biological functions. This increased density reflects greater functional convergence among the top-ranked terms and more consistent enrichment signals across clusters, thereby indicating a more robust and biologically coherent pathway landscape in the latter.

Group-wise comparisons strengthened these findings. Of note, a significant overlap was confirmed, thus indicating that patients with atherosclerosis mostly recapitulated the proteomic set observed at the whole RA group level (**Figure 3C**), whereas the effect of atherosclerosis-free group was marginal. Pathways and process analyses revealed two main clusters, one being common to whole RA group and RA with atherosclerosis group (broad immune activation and inflammation), whereas the second one was specific to the patient subset with atherosclerosis and included functionally-related signatures (**Figure 3D**). Network analysis supported this differential usage of retrieved terms between groups (**Figure 3E**).

**Figure 4:**
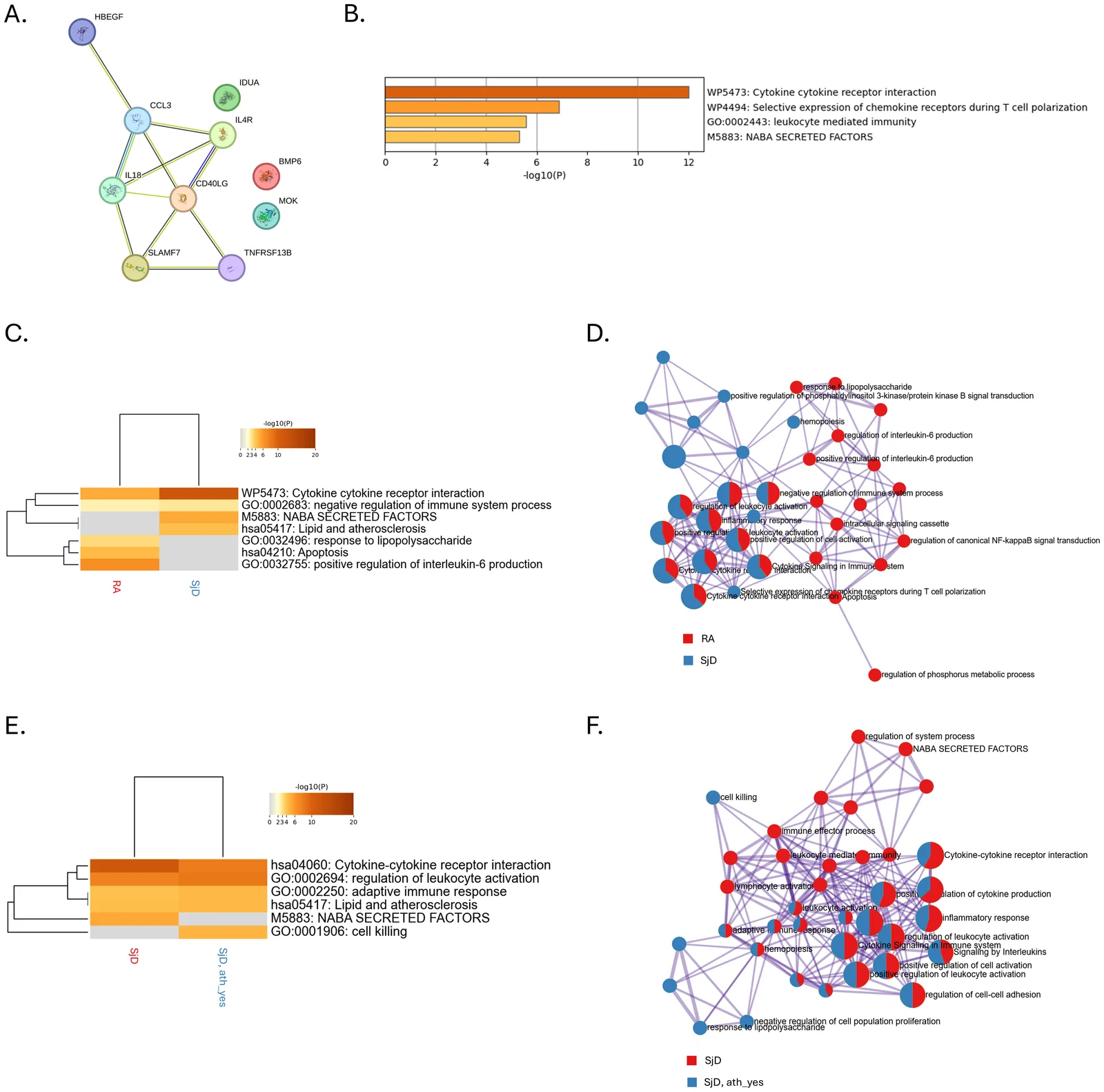
Proteomic analyses of anti-CXCR3 autoantibodies in SjD, atherosclerosis status and disease overlap. Analysis of proteomic hits associated with anti-CXCR3 antibodies in SjD (n=44) were performed. Protein-protein interaction networks **(A)** and protein and process enrichment analysis **(B)** were conducted as in the RA group (**Figure 3**). SjD and RA proteomic outputs were analyzed by heatmap **(C)** and network term layout **(D)**. Color code represents RA (red) and SjD (blue). Within SjD, group-wise comparisons among SjD (n=44), and SjD patients with atherosclerosis (n=22) were performed in heatmap **(E)** and network term layout **(F)** as conducted in the RA group (**Figure 3**). Color code represents whole SjD group (red) and SjD patients with atherosclerosis (blue).

The analysis of the SjD cohort yielded equivalent findings compared to RA. Although proteomic hits associated with anti-CXCR3 in SjD (n=10) (**Figure 4A**) slightly diverged from those in RA, pathways retrieved were overall similar (**Figure 4B**). Group-wise analyses confirmed overlap in pathway and process enrichment analyses (**Figures 4C, D**). Interestingly, most of the proteomic correlates were overall recapitulated (6/10) by the group of patients with atherosclerosis in SjD (n=22) (**Figures 4E, F**).

Altogether, these findings suggest that anti-CXCR3 antibodies were associated with proteomic signatures linked to immune activation and to apoptosis, chemotaxis, and cell adhesion in an atherosclerosis-dependent manner. This effect was consistent across systemic autoimmune conditions.

### 3.4 CXCR3 expression at systemic and tissue level in systemic autoimmunity

Next, in order to get insight into anti-CXCR3 alterations, the expression of CXCR3 in systemic autoimmunity was evaluated.

**Figure 5:**
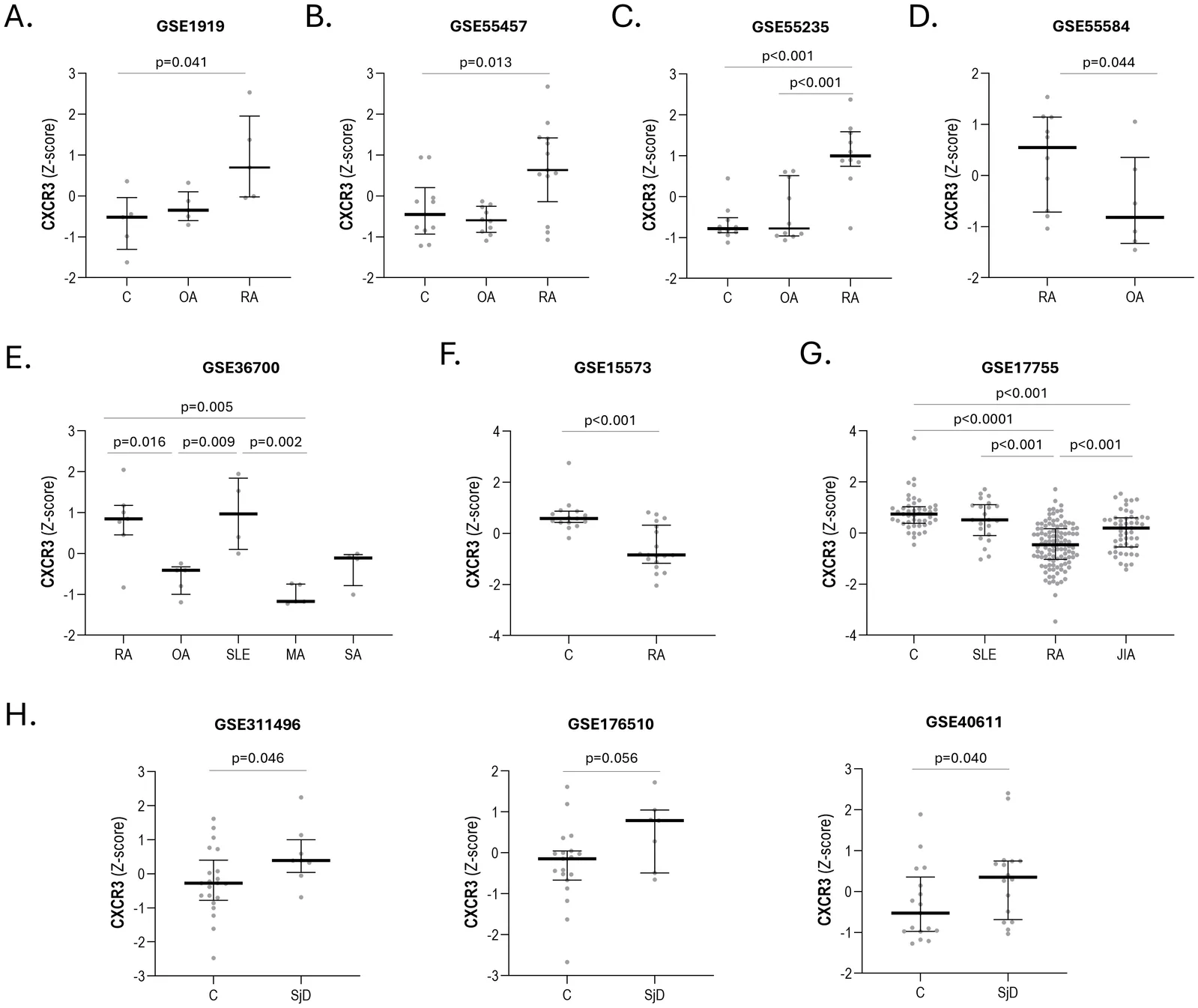
CXCR3 expression in systemic autoimmune diseases. CXCR3 expression was obtained from publicly available datasets from GEO. Datasets containing CXCR3 expression data from synovial biopsies **(A–E)** or peripheral blood **(F, G)** in RA, and target tissue (ocular and salivary gland tissue) in SjD **(H)** were analyzed. CXCR3 differential expression was confirmed by GEO2R (see Results section) within the whole dataset, and CXCR3 expression was extracted from each dataset, normalized (Z-score) and individually analyzed by conventional analyses (Kruskal-Wallis or Mann-Whitney U tests, as appropriate). Bars represent 25th percentile (lower), median and 75th percentile (upper). P-values were indicated. Group labelling was maintained as per original nomenclature. C, control; JIA, juvenile idiopathic arthritis; MA, microcrystalline arthritis; OA, osteoarthritis; RA, rheumatoid arthritis; SA, seronegative arthritis; SjD, Sjögren disease.

Data from CXCR3 gene expression both in the systemic compartment was well as in target tissue (synovium) was extracted from publicly available microarray datasets from the GEO database. Four datasets containing relevant samples for synovial tissue analysis were found. GSE1919 included gene expression data from 5 RA patients, 5 OA patients and 5 controls. A differential gene expression for CXCR3 was obtained [p(adj)=0.023], thus demonstrating an enhanced CXCR3 expression in the RA group (**Figure 5A**). Analysis of the datasets GSE55457 (13 RA, 10 OA, 10 controls) (**Figure 5B**) and GSE55235 (13 RA, 10 OA, 10 controls) (**Figure 5B**) confirmed these findings [p(adj)=0.010 and p(adj)=6.65·10^-5^, respectively]. Differences between RA (n=10) and OA (n=6) were also replicated in the GSE55584 dataset (p=0.044) (**Figure 5D**). GSE36700 also confirmed the differential CXCR3 expression at the synovial level (p(adj)=0.011) (**Figure 5E**) compared to a broader range of disease groups (7 RA, 5 OA, 4 SLE, 4 microcrystalline arthritis, 4 seronegative arthritis).

Next, CXCR3 expression in the systemic compartment was analyzed. GSE15573 revealed a differential CXCR3 gene expression [p(adj)=3.34·10^-5^] between RA (n=18) and controls (n=15) from peripheral blood mononuclear cell samples (**Figure 5F**). Similarly, results from GSE17755, containing peripheral blood samples from 112 RA patients, 22 SLE patients and 45 controls, found differences in CXCR3 expression [p(adj)=2.10·10^-3^] (**Figure 5G**). Subgroup analysis revealed diminished CXCR3 expression in RA compared to control group, and to a similar level than JIA patients (**Figure 5G**), hence confirming a specific CXCR3 downregulation in the systemic compartment in inflammatory arthritidies.

Finally, validation in SjD datasets was conducted. GSE311496 included gene expression data from ocular tissue of 8 SjD and 21 controls, and CXCR3 upregulation was confirmed (**Figure 5H**). Equivalent findings were found in conjunctiva (GSE176510) and parotid gland (GSE40611) tissue (**Figure 5H**). No datasets derived from the systemic compartment could be identified in SjD.

Collectively, analysis of multiple publicly available datasets consistently demonstrated compartment-specific alterations of CXCR3 expression in the setting of RA, namely increased CXCR3 expression at synovial tissue level, alongside a concomitant downregulation in the systemic compartment. Evidence from SjD reinforced CXCR3 upregulation in target tissues. These analyses should be interpreted with caution as derived from independent cohorts not linked to study participants.

## 4 Discussion

Although conventionally regarded as aberrant immune responses primarily driving tissue damage, the conceptualization of autoantibodies has substantially evolved over recent decades. Herein we demonstrate that autoantibodies targeting CXCR3 were altered in systemic autoimmunity. Notably, reduced anti-CXCR3 were unrelated to disease duration and already detectable in the earliest stage of the disease. Anti-CXCR3 were linked with atherosclerosis burden and related to a variety of pathogenic proteomic pathways. To the best of our knowledge, this is the first study investigating regulatory autoantibody levels in early arthritis and to demonstrate an association with atherosclerosis in autoimmunity.

Our findings consistently revealed decreased anti-CXCR3 levels in RA and SjD, in line with prior evidence in SjD (16) and similar evidence suggested for other regulatory antibodies in vasculitis (17). This observation points to a common immunoregulatory pathway involving the CXCR3 axis across autoimmune conditions, hence strengthening its biological relevance. This aligns with the shared autoantibody signatures observed in neurological conditions (18). Notably, the absence of associations with disease features, along with the findings from CSA, reinforce a potential role in disease initiation and triggering, and argue against these being secondary to chronic inflammation or an epiphenomenon. This is in line with their role as natural components of the immune system (19). Altogether, these observations challenge the current view of autoantibodies in autoimmunity, and promote a shift from a purely pathogenic paradigm toward a “double-edge sword” framework, where antibodies may also constitute an endogenous homeostatic system. The inverse association with poor therapeutic outcomes is in line with this interpretation, and mirrors similar results reported in ANCA-associated vasculitis (20). Similarly, lower anti-CXCR3 antibodies were also predictors of lung function deterioration in SSc (9). Although initially counterintuitive, observing decreased autoantibodies in autoimmunity is not unprecedented (21). Additionally, a broad repertoire of IgG autoantibodies can be detected in the general population (22), with intrinsic properties of common autoantigens having been demonstrated, and contrasting with disease-related autoantibodies. Integration into antibody network landscapes is imperative to elucidate functional correlates of these findings. In this context, the concept of antibodyome emerges as a powerful tool to dissect mechanisms underlying altered levels of regulatory antibodies and to establish functional significance in autoimmunity.

A major breakthrough from our study was the robust association between anti-CXCR3 antibody levels and atherosclerosis in autoimmunity, which represent an unprecedented observation with pathological relevance. Reduced anti-CXCR3 levels were consistently associated with atherosclerosis occurrence and extent both in RA and SjD, and it was independent of traditional CV risk factors and treatment exposure. The validation of this result in a cohort of established RA patients with a history of CVD provides valuable insight in a two-fold manner. First, as it confirms this association with clinical overt CVD. Second, as it validates this association in a composite definition of CVD including non-ischemic manifestations, thereby broadening the clinical spectrum of relevance. Mechanistic evidence linking CXCR3+ T-cells and cardiac remodeling further support this observation (23). Although a potential role as diagnostic marker had been proposed (24, 25), their contribution as clinical biomarker remained insufficiently explored. By conducting a comprehensive analysis of their incremental value, our data proved that anti-CXCR3 can be not only independent predictors but also yielded significant added value for risk stratification. Moreover, they can be combined with existing instruments for risk stratification, thereby addressing a major unmet need in the research agenda (26, 27). Finally, recent data from a large registry has unveiled a positive association between anti-CXCR3 antibodies and CV risk in the general population (8), which contrast with our findings. However, these discrepancies should be interpreted with caution, particularly in the light of the autoimmune background. Accumulating evidence has demonstrated that autoimmunity not only represents an additional risk factor itself [reviewed in (28)] but also substantially modifies the relative contribution of traditional CV risk factors (29, 30). Therefore, distinct mechanistic circuits operating may be key to reconcile these findings. Functional differences in antibodies between these two populations cannot be ruled out.

Although certain autoantibodies have been found to be decreased in atherosclerosis, mostly involved in clearance, the participation of regulatory targeting GPCRs calls for a novel framework. Experimental CXCR3 blockade or inactivation has been shown to dampen atherosclerosis progression (31), which is consistent with the negative association of anti-CXCR3 levels herein suggested. Mechanistic studies, including biological activity, receptor-binding properties, effects on signaling or ability to modulate immune-cell migration, are needed to confirm whether this informs a protective effect in this setting. CXCR3 is central to Th1-polarized lymphocyte migration toward sites of inflammation, and its ligands are strongly expressed in both inflamed synovium (32, 33) and salivary glands (34), especially during disease exacerbation. Therefore, reduced circulating anti-CXCR3 autoantibodies may rather reflect a selective accumulation within target tissues, thus leading to a loss of endogenous mechanisms that normally restrain chemokine signaling and immune cell recruitment in the systemic compartment. An agonistic effect of anti-CXCR3 on immune cell migration in autoimmunity has been suggested (9). Given that anti-CXCR3 antibodies may interfere with cell trafficking, decreased levels may in turn favor enhanced lymphocyte and monocyte transendothelial migration in the vasculature, thereby promoting atherosclerosis development. The consistent differential CXCR3 expression between synovial and systemic compartment in RA and SjD strongly supports this notion, also in line with the Th1 profile observed at the synovial level (35). This hypothesis may be also in line with the occurrence of shared mechanisms between joint inflammation and atherosclerosis development (36). Notably, it may provide a functional insight into the connection between poor therapeutic outcomes and CV risk (37, 38), also in accordance with our data. Of note, these findings provide robust observational evidence toward previous hypothesis (39). Taken together, all these data herein reported along with literature support consistently shed new light into delineating anti-CXCR3 involvement in atherosclerosis in autoimmunity.

Alternatively, low anti-CXCR3 antibodies might reflect enhanced endothelium binding. CXCR3 upregulation in endothelial cells upon inflammation has been reported, especially in unstable plaques (8), and anti-CXCR3 antibodies may therefore bind within the lesion and become sequestered or consumed (40). This ‘sink’ effect would in turn mean less systemic coverage of CXCR3+ cells, thus enabling a more efficient CXCR3-dependent homing to sites of arterial inflammation. The associations with plaque characteristics in our as well as the enrichment of cell adhesion and chemotaxis pathways in our proteomic platform may support this hypothesis. Interestingly, increased CXCR3+ T-cells in atherosclerosis lesions have been described in lupus (41). This is also in line with the proposed role of anti-GPCRs antibodies as modulators of vascular-immune interface (5). Further evidence validating CXCR3 expression data in endothelium and/or atherosclerosis plaques in autoimmune conditions and cell migration experiments are warranted to prove this hypothesis.

Additionally, regulatory autoantibodies can also exert their effects via receptor ligation and internalization (6), leading to tonic desensitization, thereby enhancing chemotactic transendothelial migration into plaques. Other mechanisms such as antibody consumption by complement activation may be ruled out, at least in part, according to our findings, also in line with Fab-dependency and IgG subclass usage (6). Finally, antibody production may differ between patients and controls, so affinities and functions may also be considered. In sum, decreased anti-CXCR3 regulatory antibodies could plausibly favor enhanced CXCR3-dependent leukocyte recruitment into plaques, or altered receptor desensitization. These mechanisms, or a combination of thereof, may potentially aggravate atherosclerosis progression in autoimmunity. However, because functional assays were not performed in our study, our data support an association between reduced circulating anti-CXCR3 antibodies and atherosclerosis in autoimmunity, but whether these antibodies act as agonists, antagonists, or neutral regulators of CXCR3 signaling cannot be established and should be interpreted with caution as proposed hypothesis. Mechanistic validation remains to be established in future studies. Elucidating the exact functional phenotype of these antibodies and their compartmentalization, and passive transfer experiments in autoimmune and non-autoimmune models are warranted to provide experimental confirmation. Finally, the diversity of functional pathways retrieved for anti-CXCR3 underscores the central role of GPCRs signaling in driving disease mechanisms and highlight the contribution of non-traditional CV risk mechanisms. Delineating individual GPCRs signatures is imperative to explain individual susceptibility and encourages integrative analysis to decipher functional networks.

The characterization of anti-CXCR3 antibodies in relation to clinical outcomes in RA and SjD paves the way for a comprehensive investigation of anti-GPCRs antibodies in the broader context of autoimmunity. Given the central role of GPCRs in regulating a plethora of biological processes, these antibodies may shed new light into systemic manifestations, such as neurological outcomes including fatigue, dysautonomia or cognitive dysfunction (5), which represent critical determinants of quality of life. Furthermore, deciphering regulatory antibody networks in these conditions will likely inform complex, multi-system interactions underlying autoimmune pathogenesis at the systemic level (7). Recent data from other clinical scenarios, including limited evidence from SjD, are reassuring in this regard (16). Whether disturbed antibody networks may represent a viable therapeutic target in autoimmunity is yet to be determined. Although our findings failed to exhibit significant changes in antibody levels upon TNFi exposure, it is conceivable that these fluctuations inform changes in antibody networks that may be of biological and clinical relevance, as reported in other complex systems (42).

This study comes with certain limitations that should be noted. First, although regulatory properties for anti-GPCR antibodies have been demonstrated, receptor binding, signaling effects, subclass function, or cell-migration activity were not specifically assessed in our study; therefore, the functional phenotype of anti-CXCR3 antibodies in our cohort remains unknown. Future studies encompassing functional characterization of anti-CXCR3 antibodies in RA and SjD are needed to elucidate their protective role. On the other hand, although shared mechanisms were retrieved in RA and SjD, whether this may apply to other autoimmune conditions also hallmarked by premature atherosclerosis burden, such as lupus or SSc, is yet to be explored. Furthermore, validation of CXCR3 expression came from independent cohorts, to provide indirect, hypothesis-generating context regarding CXCR3 expression patterns, and they were not linked to the individuals included in the present serological study. Future paired studies are awaited to validate compartment-specific alterations, especially in SjD. Cross sectional design, along with the lack of functional characterization, prevented the establishment of causality of mechanistic role of anti-CXCR3 antibodies. Moreover, a potential residual confounding in the association between anti-CXCR3 and atherosclerosis cannot be totally excluded, especially in relation to cumulative inflammatory burden. Longitudinal studies with time-series analyses might be appropriate to rule out this possibility. Additionally, subgroup analysis (such as CSA or TNFi) should be considered as exploratory and require replication in larger samples and longer observation timeframes. Finally, prospective trials to assess added value in hard clinical endpoints (i.e. MACE) are warranted.

In conclusion, altered anti-CXCR3 antibody levels may represent a common hallmark of autoimmunity. Diminished anti-CXCR3 could yield new insight into disease pathogenesis, clinical implications and broad antibody biology in rheumatic diseases. Clinical translation of anti-CXCR3 antibodies hold promise to improve risk stratification beyond traditional scores. Collectively, these findings align with the emerging concept that components of the regulatory autoantibody repertoire can exert immunomodulatory functions and that network-wide disruptions may contribute to immune dysregulation and clinical outcomes in rheumatic autoimmune diseases. This study opens new avenues toward system-level approaches integrating multi-layered characterization of regulatory antibody networks to improve our understanding of systemic autoimmunity and identifying potential therapeutic targets, and inform functional studies to cover this knowledge gap. A reframing of the current concept of autoantibodies in autoimmunity within this expanded, regulatory framework is encouraged.

## Data Availability

The raw data supporting the conclusions of this article will be made available by the authors, without undue reservation.

## References

[1] Porsch F Binder CJ . Autoimmune diseases and atherosclerotic cardiovascular disease. Nat Rev Cardiol. (2024) 21:780–807. doi: 10.1038/s41569-024-01045-7 38937626

[2] Buch MH Mallat Z Dweck MR Tarkin JM O’Regan DP Ferreira V . Current understanding and management of cardiovascular involvement in rheumatic immune-mediated inflammatory diseases. Nat Rev Rheumatol. (2024) 20:614–34. doi: 10.1038/s41584-024-01149-x 39232242

[3] Porsch F Mallat Z Binder CJ . Humoral immunity in atherosclerosis and myocardial infarction: from B cells to antibodies. Cardiovasc Res. (2021) 17 (13):2544–62. doi: 10.1093/cvr/cvab285 34450620

[4] Deroissart J Binder CJ Porsch F . Role of antibodies and their specificities in atherosclerotic cardiovascular disease. Arterioscler Thromb Vasc Biol. (2024) 44:2154–68. doi: 10.1161/ATVBAHA.124.319843 39114917

[5] Cabral-Marques O Schimke LF Moll G Filgueiras IS Nóbile AL Adri AS . Advancing research on regulatory autoantibodies targeting GPCRs: insights from the 5th international symposium. Autoimmun Rev. (2025) 24:103855. doi: 10.1016/j.autrev.2025.103855 40543860

[6] Yu X Wax J Riemekasten G Petersen F . Functional autoantibodies: definition, mechanisms, origin and contributions to autoimmune and non-autoimmune disorders. Autoimmun Rev. (2023) 22:103386. doi: 10.1016/j.autrev.2023.103386 37352904

[7] Cabral-Marques O Riemekasten G . Functional autoantibodies targeting G protein-coupled receptors in rheumatic diseases. Nat Rev Rheumatol. (2017) 13:648–56. doi: 10.1038/nrrheum.2017.134 28855694

[8] Müller FS Aherrahrou Z Grasshoff H Heidorn MW Humrich JY Johanson L . Autoantibodies against the chemokine receptor 3 predict cardiovascular risk. Eur Heart J. (2023) 44:4935–49. doi: 10.1093/eurheartj/ehad666 37941454 PMC10719496

[9] Weigold F Günther J Pfeiffenberger M Cabral-Marques O Siegert E Dragun D . Antibodies against chemokine receptors CXCR3 and CXCR4 predict progressive deterioration of lung function in patients with systemic sclerosis. Arthritis Res Ther. (2018) 20:52. doi: 10.1186/s13075-018-1545-8 29566745 PMC5863842

[10] Aletaha D Neogi T Silman AJ Funovits J Felson DT Bingham CO . 2010 rheumatoid arthritis classification criteria: an American College of Rheumatology/European League Against Rheumatism collaborative initiative. Ann Rheum Dis. (2010) 69:1580–8. doi: 10.1136/ard.2010.138461 20699241

[11] van Steenbergen HW Aletaha D Beaart-van de Voorde LJJ Brouwer E Codreanu C Combe B . EULAR definition of arthralgia suspicious for progression to rheumatoid arthritis. Ann Rheum Dis. (2017) 76:491–6. doi: 10.1136/annrheumdis-2016-209846 27991858

[12] Rodríguez-Carrio J Alperi-López M López P Ballina-Garciá FJ Abal F Suárez A . Antibodies to high-density lipoproteins are associated with inflammation and cardiovascular disease in rheumatoid arthritis patients. Trans Res. (2015) 166:529–39. doi: 10.1016/j.trsl.2015.07.004 26279255

[13] Vitali C Bombardieri S Jonsson R Moutsopoulos HM Alexander EL Carsons SE . Classification criteria for Sjögren’s syndrome: a revised version of the European criteria proposed by the American-European Consensus Group. Ann Rheum Dis. (2002) 61:554–8. doi: 10.1136/ard.61.6.554 12006334 PMC1754137

[14] Miranda-Prieto D Alperi-López M Pérez-Álvarez Á Alonso-Castro S Suárez A Rodríguez-Carrio J . Higher frequencies of age-associated B-cells in early arthritis are linked to atherosclerosis and immune circuits—a potential role as a biomarker for risk stratification. Rheumatology. (2025) 64:5921–31. doi: 10.1093/rheumatology/keaf318 40478777

[15] Lundberg M Eriksson A Tran B Assarsson E Fredriksson S . Homogeneous antibody-based proximity extension assays provide sensitive and specific detection of low-abundant proteins in human blood. Nucleic Acids Res. (2011) 39:e102–2. doi: 10.1093/nar/gkr424 21646338 PMC3159481

[16] Yue X Deng F Chen J Yin J Zheng J Chen Y . Autoantibodies against C5aR1, C3aR1, CXCR3, and CXCR4 are decreased in primary Sjogren’s syndrome. Mol Immunol. (2021) 131:112–20. doi: 10.1016/j.molimm.2020.12.027 33446393

[17] Klapa S Müller A Koch A Heidecke H Kähler W Junker J . Decreased endothelin receptor A autoantibody levels are associated with early ischaemic events in patients with giant-cell arteritis. Ann Rheum Dis. (2019) 78:1443–4. doi: 10.1136/annrheumdis-2019-215341 31186255

[18] Nakanishi Usuda J Nóbile AL Nery do Vale FY Corrêa YLG Adri AS Nava RG . Integrative analysis reveals the autoantibodyome neuroimmune signature of neurodegeneration. iScience. (2026) 29:114781. doi: 10.1016/j.isci.2026.114781 41704760 PMC12907111

[19] Cabral-Marques O Marques A Giil LM De Vito R Rademacher J Günther J . GPCR-specific autoantibody signatures are associated with physiological and pathological immune homeostasis. Nat Commun. (2018) 9:5224. doi: 10.1038/s41467-018-07598-9 30523250 PMC6283882

[20] Klapa S Müller A Arnold S Koch A Staehle A Kaehler W . Decreased concentrations of C5a complement receptor antibodies are associated with relapse in eosinophilic granulomatosis with polyangiitis (EGPA). RMD Open. (2025) 11:e005323. doi: 10.1136/rmdopen-2024-005323 40050040 PMC11887279

[21] Nagele EP Han M Acharya NK DeMarshall C Kosciuk MC Nagele RG . Natural IgG autoantibodies are abundant and ubiquitous in human sera, and their number is influenced by age, gender, and disease. PloS One. (2013) 8:e60726. doi: 10.1371/journal.pone.0060726 23589757 PMC3617628

[22] Shome M Chung Y Chavan R Park JG Qiu J LaBaer J . Serum autoantibodyome reveals that healthy individuals share common autoantibodies. Cell Rep. (2022) 39:110873. doi: 10.1016/j.celrep.2022.110873 35649350 PMC9221390

[23] Laroumanie F Douin-EChinard V Pozzo J Lairez O Tortosa F Vinel C . CD4 ^+^ T cells promote the transition from hypertrophy to heart failure during chronic pressure overload. Circulation. (2014) 129:2111–24. doi: 10.1161/CIRCULATIONAHA.113.007101 24657994

[24] Ernst D Widera C Weiberg D Derlin T Ahrenstorf G Sogkas G . Beta-1-adrenergic receptor antibodies in acute coronary syndrome: is less sometimes more? Front Cardiovasc Med. (2018) 5. doi: 10.3389/fcvm.2018.00170 30619882 PMC6305491

[25] Ernst D Westerbergh J Sogkas G Jablonka A Ahrenstorf G Schmidt RE . Lowered anti-beta1 adrenergic receptor antibody concentrations may have prognostic significance in acute coronary syndrome. Sci Rep. (2019) 9:14552. doi: 10.1038/s41598-019-51125-9 31601947 PMC6787077

[26] Agca R Heslinga SC Rollefstad S Heslinga M McInnes IB Peters MJL . EULAR recommendations for cardiovascular disease risk management in patients with rheumatoid arthritis and other forms of inflammatory joint disorders: 2015/2016 update. Ann Rheum Dis. (2017) 76:17–28. doi: 10.1136/annrheumdis-2016-209775 27697765

[27] Drosos GC Vedder D Houben E Boekel L Atzeni F Badreh S . EULAR recommendations for cardiovascular risk management in rheumatic and musculoskeletal diseases, including systemic lupus erythematosus and antiphospholipid syndrome. Ann Rheum Dis. (2022) 81:768–79. doi: 10.1136/annrheumdis-2021-221733 35110331

[28] England BR Thiele GM Anderson DR Mikuls TR . Increased cardiovascular risk in rheumatoid arthritis: mechanisms and implications. BMJ. (2018) 361:k1036. doi: 10.1136/bmj.k1036 29685876 PMC6889899

[29] Gonzalez A Maradit Kremers H Crowson CS Ballman KV Roger VL Jacobsen SJ . Do cardiovascular risk factors confer the same risk for cardiovascular outcomes in rheumatoid arthritis patients as in non-rheumatoid arthritis patients? Ann Rheum Dis. (2008) 67:64–9. doi: 10.1136/ard.2006.059980 17517756

[30] Crowson CS Rollefstad S Ikdahl E Kitas GD van Riel PLCM Gabriel SE . Impact of risk factors associated with cardiovascular outcomes in patients with rheumatoid arthritis. Ann Rheum Dis. (2018) 77:48–54. doi: 10.1136/annrheumdis-2017-211735 28877868 PMC5726925

[31] Ngwenyama N Salvador AM Velázquez F Nevers T Levy A Aronovitz M . CXCR3 regulates CD4+ T cell cardiotropism in pressure overload–induced cardiac dysfunction. JCI Insight. (2019) 4(7):e125527. doi: 10.1172/jci.insight.125527 30779709 PMC6483643

[32] Ruschpler P Lorenz P Eichler W Koczan D Hänel C Scholz R . High CXCR3 expression in synovial mast cells associated with CXCL9 and CXCL10 expression in inflammatory synovial tissues of patients with rheumatoid arthritis. Arthritis Res Ther. (2003) 5:R241. doi: 10.1186/ar783 12932287 PMC193722

[33] Patel DD Zachariah JP Whichard LP . CXCR3 and CCR5 ligands in rheumatoid arthritis synovium. Clin Immunol. (2001) 98:39–45. doi: 10.1006/clim.2000.4957 11141325

[34] Kim JW Ahn MH Jung JY Suh CH Han JH Kim HA . Role of chemokines CXCL9, CXCL10, CXCL11, and CXCR3 in the serum and minor salivary gland tissues of patients with Sjögren’s syndrome. Clin Exp Med. (2024) 24:133. doi: 10.1007/s10238-024-01401-4 38900301 PMC11189950

[35] Iwata S Zhang M Hajime M Ohkubo N Sonomoto K Torimoto K . Pathological role of activated mTOR in CXCR3+ memory B cells of rheumatoid arthritis. Rheumatology. (2021) 60:5452–62. doi: 10.1093/rheumatology/keab229 33693564

[36] Weber BN Giles JT Liao KP . Shared inflammatory pathways of rheumatoid arthritis and atherosclerotic cardiovascular disease. Nat Rev Rheumatol. (2023) 19:417–28. doi: 10.1038/s41584-023-00969-7 37231248 PMC10330911

[37] Dixon WG Watson KD Lunt M Hyrich KL Silman AJ Symmons DPM . Reduction in the incidence of myocardial infarction in patients with rheumatoid arthritis who respond to anti–tumor necrosis factor α therapy: results from the British Society for Rheumatology Biologics Register. Arthritis Rheum. (2007) 56:2905–12. doi: 10.1002/art.22809 17763428 PMC2435427

[38] Solomon DH Reed GW Kremer JM Curtis JR Farkouh ME Harrold LR . Disease activity in rheumatoid arthritis and the risk of cardiovascular events. Arthritis Rheumatol. (2015) 67:1449–55. doi: 10.1002/art.39098 25776112 PMC4446181

[39] Riemekasten G Petersen F Heidecke H . What makes antibodies against G protein-coupled receptors so special? A novel concept to understand chronic diseases. Front Immunol. (2020) 11. doi: 10.3389/fimmu.2020.564526 33384684 PMC7770155

[40] Szentes V Gazdag M Szokodi I Dézsi CA . The role of CXCR3 and associated chemokines in the development of atherosclerosis and during myocardial infarction. Front Immunol. (2018) 9. doi: 10.3389/fimmu.2018.01932 30210493 PMC6119714

[41] Clement M Charles N Escoubet B Guedj K Chauveheid MP Caligiuri G . CD4+CXCR3+ T cells and plasmacytoid dendritic cells drive accelerated atherosclerosis associated with systemic lupus erythematosus. J Autoimmun. (2015) 63:59–67. doi: 10.1016/j.jaut.2015.07.001 26183767

[42] Rodríguez-Carrio J Alperi-López M López P Ballina-García FJ Suárez A . Heterogeneity of the type I interferon signature in rheumatoid arthritis: a potential limitation for its use as a clinical biomarker. Front Immunol. (2018) 8. doi: 10.3389/fimmu.2017.02007 29387065 PMC5775969

