# Supplementary material for: Reduced circulating anti-CXCR3 antibodies as a common hallmark bridging systemic autoimmunity and atherosclerosis
Source: Front Immunol. 2026 Sep 3;17:1937388. doi: 10.3389/fimmu.2026.1937388 (PMC13582333; doi:10.3389/fimmu.2026.1937388)
Supplement: Supplementary file 1 [file Supplementaryfile1.docx]

**SUPPLEMENTARY MATERIALS**

Supplementary Material and Methods

Supplementary Tables

**SUPPLEMENTARY MATERIAL AND METHODS**

**Study participants**

Early RA patients fulfilling 2010 ACR/EULAR classification criteria [1] were recruited at disease diagnosis from the early arthritic clinic of the Department of Rheumatology at Hospital Universitario Central de Asturias (HUCA). Early RA patients were not exposed to any disease-modifying antirheumatic drugs (DMARDs) at recruitment, and disease duration can be considered zero due to recruitment at diagnosis [2]. A complete clinical examination was performed during the clinical appointment, including Disease Activity Score 28-joints and Health Assessment Questionnaire (HAQ) calculations. Traditional CV risk factors (dyslipidaemia, hypertension, diabetes, smoking habits and obesity) were obtained from the medical records and defined in compliance with national guidelines. A group of individuals with clinically suspect arthralgia (CSA) [3] were also recruited from the same clinic. A separate cohort of long-lasting, established RA patients recruited from the same institution was included as validation cohort [4]. Established RA was defined as disease duration >1 year [5,6]. Additionally, a group of 13 biological-naïve RA patients was prospectively followed upon TNF blockade initiation and a blood sample was obtained before (baseline) and 3 months after anti-TNF therapy. Clinical response after TNF blockade was evaluated using disease activity cut offs and timeframes (3 months) according to EULAR criteria [7]. A priori sample size calculations using RA data relative to DMARDs exposure (alpha 0.050, sigma 14.76, and delta 16.12) estimated a sample size of 11 individuals with paired data to achieve a power of 0.80 [8]. Healthy controls (HC) were recruited among age- and sex- matched healthy volunteers from the same population.

Furthermore, a cohort of SjD patients fulfilling American-European Consensus Group criteria [9] was recruited from the Department of Internal Medicine (HUCA). These patients undergone a complete clinical examination, including Sjögren's syndrome disease activity index (ESSDAI) calculation, and traditional CV risk assessment as previously mentioned.

Fasting blood samples were collected from all individuals by venepuncture in EDTA-containing tubes. Blood samples were immediately transferred to the laboratory and processed within less than 2 hours. Serum samples were stored at -80ºC until experimental procedures. Conventional blood biochemical, including C-reactive Protein (CRP) and Erythrocyte Sedimentation Rate (ESR) measurements, lipid analyses and complete blood counts were carried out in all individuals.

The study was approved by the local institutional review board (Comité de Ética de Investigación con Medicamentos del Principado de Asturias) in compliance with the Declaration of Helsinki (reference CEImPA 2021.126). All study subjects gave written informed consent.

**Echocardiographic study**

Subclinical atherosclerosis was assessed by Doppler ultrasound at the sonography laboratory (Department of Neurology, HUCA) online in B-mode using a Toshiba Aplio XG device (Toshiba American Medical Systems) by an experienced user, as previously described [10]. The carotid intima-media thickness (cIMT) was bilaterally measured following the “Mannheim Carotid Intima-Media Thickness Consensus 5 (2004-2006) [11], and atherosclerotic plaque burden (presence and extent) were defined according to established definitions as previously described [11]. Plaque vulnerability status was evaluated by the ultrasound characterization of the plaques (border profile, echo-density, calcification, and burden), and these were classified as “low” or “high” risk [12].

**Anti-CXCR3 assessment**

Levels of anti-CXCR3 IgG antibodies were quantified in serum samples (diluted 1/100) using a commercial assay (EIA for Quantitative Determination of anti-CXCR3-Antibodies, CellTrend), following the instructions provided by the manufacturer. This kit has been implemented to maintain the native receptor conformation [13–15]. ensuring that functional autoantibodies recognize the target effectively but do not distinguish downstream functional effects [16]. Samples were assayed in a group-match manner per plate, to control for potential batch effects. Positive controls were included in each plate, as a quality control. Values were expressed as U/ml, according to standard curves. Detection limit was 2.5 U/ml. Intra- and inter-assay coefficient of variation were 6.1% and 8.0%, respectively.

**Cytokine analyses**

Interleukin (IL)-6, Tumour Necrosis Factor (TNF), Interferon gamma (IFNγ), IL-1b, IL-23, IL-12, IL-33, IL-10, IL-17, IL-8, and IL-18 serum levels were measured by a predefined multiplex assay (Human Inflammation Panel 1, LEGENDplex, BioLegend). Detection limits were 2.68 pg/ml, 2.90 pg/ml, 2.68 pg/ml, 3.17 pg/ml, 4.39 pg/ml, 19.53 pg/ml, 3.66 pg/ml, 0.73 pg/ml, 2.92 pg/ml, and 3.90 pg/ml, respectively. The serum levels of IFNα were quantified using a Cytometric Bead Array Flex Set (BD). Detection limit was 1.25 pg/ml, A Proliferation -Inducing Ligand (APRIL), B-cell Activating Factor (BAFF) and IL-21 serum levels were quantified using ELISA kits (Invitrogen, ThermoFisher). Detection limits were 0.40 ng/ml, 0.13 ng/ml and 20 pg/ml, respectively.

**Proteomic platform**

Serum proteomics were carried out using a high-throughput analysis by means of the Proximity Extension Assay [17]. In brief, a predefined panel of 92 protein species related to CV was measured using Olink technology (Cardiovascular Panel II) (Olink Bioscience, Sweden).

**Statistical analysis**

Variables were summarised as median (interquartile range) or mean ± standard deviation, or *n* (%), as appropriate.

Differences among groups were evaluated by Mann-Whitney *U*, Kruskal–Wallis tests with Dunn-Bonferroni correction for multiple comparisons, or Wilcoxon paired tests, according to the distribution of the variables. Correlations were assessed by Spearman ranks’ test. The associations between anti-CXCR3 levels and atherosclerosis occurrence were analyzed by univariate and multivariate logistic regression adjusted by confounders, namely traditional CV risk factors defined according to guidelines (sex, age, hypertension, diabetes, smoking and dyslipidemia) and treatment exposure and disease duration, when applicable (only for established disease). Adjustment for confounders in multivariate models was performed systematically and in an unsupervised manner. Negative binomial regression models were used when plaque number was modeled as outcome variable. Odds ratio (OR) and 95% confidence intervals (CI) were calculated. Anti-CXCR3 IgG levels were added to the mSCORE by using tertiles observed in the HC population as cut-offs, with scorings been given as follows [18]: 0 to T1: +2, T1 to T2: +1, T2 to T3: +0. These points were added to the mSCORE values to generate the modified algorithm (mSCORE + anti-CXCR3). The performance on risk stratification was evaluated by classification (sensitivity, specificity, accuracy), discrimination (AUC ROC), correlation (Matthews’ correlation coefficient), and goodness of fit (Hosmer-Lemeshow test) statistics. Differences between ROC values were compared by De Long test. Net Reclassification Improvement (NRI) was used to compare the effect of risk reclassification of the modified algorithm.

Protein hits associated with anti-CXCR3 levels were identified by correlation tests after adjusting for multiple for multiple comparisons as previously described [18]. Protein-protein interactions were assessed by the STRING database [19], and pathway enrichment analyses were evaluated by pathway using Metascape [20] using KEGG pathway, GO biological processes, Reactome Gene Sets, Canonical Pathways, CORUM, and PANTHER Pathway as ontology sources (threshold: kappa=3). Term network analyses were performed under Cytoscape [21]. Group-wise comparisons were performed under Metascape and visualized using circus plots, and heatmap of enriched terms with hierarchical clustering.

Gene expression datasets were downloaded from the publicly available National Center for Biotechnology Information (NCBI) Gene Expression Omnibus (GEO) repository [22]. For validation, differential CXCR3 expression was evaluated using the GEO2R tool and FDR-adjusted p-values (p(adj)) were computed. Then, profile data expression was downloaded, normalized (Z-score), and analyzed by conventional tests across experimental groups on each dataset.

A p-value <0.050 was considered as statistically significant. Statistical analyses were carried out under SPSS v. 27, PS Power and Sample Size Calculations v. 3.0, R v.4.1.3 and GraphPad Prism 8.0.

**References**

1.	Aletaha D, Neogi T, Silman AJ, Funovits J, Felson DT, Bingham CO, et al. 2010 rheumatoid arthritis classification criteria: an American College of Rheumatology/European League Against Rheumatism collaborative initiative. Ann Rheum Dis. 2010;69:1580–8. doi:10.1136/ard.2010.138461 PubMed PMID: 20699241.

2.	Raza K, Saber TP, Kvien TK, Tak PP, Gerlag DM. Timing the therapeutic window of opportunity in early rheumatoid arthritis: proposal for definitions of disease duration in clinical trials. Ann Rheum Dis. 2012;71:1921–3. doi:10.1136/annrheumdis-2012-201893

3.	van Steenbergen HW, Aletaha D, Beaart-van de Voorde LJJ, Brouwer E, Codreanu C, Combe B, et al. EULAR definition of arthralgia suspicious for progression to rheumatoid arthritis. Ann Rheum Dis. 2017;76:491–6. doi:10.1136/annrheumdis-2016-209846

4.	Rodríguez-Carrio J, Alperi-López M, López P, Ballina-Garciá FJ, Abal F, Suárez A. Antibodies to high-density lipoproteins are associated with inflammation and cardiovascular disease in rheumatoid arthritis patients. Translational Research. 2015;166. doi:10.1016/j.trsl.2015.07.004

5.	Tak PP. Is early rheumatoid arthritis the same disease process as late rheumatoid arthritis? Best Pract Res Clin Rheumatol. 2001;15:17–26. doi:10.1053/berh.2000.0123

6.	Horton SC, Walsh CAE, Emery P. Established rheumatoid arthritis: Rationale for best practice – Physicians’ perspective of how to realise tight control in clinical practice. Best Pract Res Clin Rheumatol. 2011;25:509–21. doi:10.1016/j.berh.2011.10.012

7.	Van Gestel AM, Anderson JJ, Van Riel PLCM, Boers M, Haagsma CJ, Rich B, et al. ACR and EULAR improvement criteria have comparable validity in rheumatoid arthritis trials. Journal of Rheumatology. 1999;26:705–11. PubMed PMID: 10090187.

8.	Dupont WD, Plummer WD. Power and sample size calculations. Control Clin Trials. 1990;11:116–28. doi:10.1016/0197-2456(90)90005-M

9.	Vitali C, Bombardieri S, Jonsson R, Moutsopoulos HM, Alexander EL, Carsons SE, et al. Classification criteria for Sjögren’s syndrome: a revised version of the European criteria proposed by the American-European Consensus Group. Ann Rheum Dis. 2002;61:554–8. PubMed PMID: 12006334.

10.	Miranda-Prieto D, Alperi-López M, Pérez-Álvarez ÁI, Alonso-Castro S, Suárez A, Rodríguez-Carrio J. Higher frequencies of age-associated B-cells in early arthritis are linked to atherosclerosis and immune circuits—a potential role as a biomarker for risk stratification. Rheumatology. 2025;64:5921–31. doi:10.1093/rheumatology/keaf318

11.	Touboul PJ, Hennerici MG, Meairs S, Adams H, Amarenco P, Bornstein N, et al. Mannheim Carotid Intima-Media Thickness Consensus (2004–2006). Cerebrovascular Diseases. 2007;23:75–80. doi:10.1159/000097034

12.	Picano E, Paterni M. Ultrasound Tissue Characterization of Vulnerable Atherosclerotic Plaque. Int J Mol Sci. 2015;16:10121–33. doi:10.3390/ijms160510121

13.	van der Wouden KE, Ahmed S, van Oostveen WM, Hoekstra EM, Liem SIE, Huizinga TWJ, et al. Autoantibodies directed against the angiotensin II type 1 receptor and the endothelin-1 type A receptor in patients with systemic sclerosis. RMD Open. 2025;11:e005787. doi:10.1136/rmdopen-2025-005787

14.	Ernst D, Westerbergh J, Sogkas G, Jablonka A, Ahrenstorf G, Schmidt RE, et al. Lowered anti-beta1 adrenergic receptor antibody concentrations may have prognostic significance in acute coronary syndrome. Sci Rep. 2019;9:14552. doi:10.1038/s41598-019-51125-9 PubMed PMID: 31601947.

15.	Schmitz B, Garbsch R, Schäfer H, Bär C, Chatterjee S, Riemekasten G, et al. Autonomic dysfunction and vasoregulation in long COVID-19 are linked to anti-GPCR autoantibodies. Journal of Allergy and Clinical Immunology. 2026;157:722-738.e7. doi:10.1016/j.jaci.2025.10.034

16.	Azcue N, Prada A, Del Pino R, Acera M, Fernández-Valle T, Ayo-Mentxakatorre N, et al. Involvement of autoantibodies against G protein-coupled receptors in post-COVID condition and Chronic Fatigue Syndrome. Sci Rep. 2026;16:20609. doi:10.1038/s41598-026-49131-9

17.	Lundberg M, Eriksson A, Tran B, Assarsson E, Fredriksson S. Homogeneous antibody-based proximity extension assays provide sensitive and specific detection of low-abundant proteins in human blood. Nucleic Acids Res. 2011;39:e102–e102. doi:10.1093/nar/gkr424

18.	Rodríguez-Carrio J, Alperi-López M, López P, Pérez-Álvarez ÁI, Robinson GA, Alonso-Castro S, et al. Humoral responses against HDL are linked to lipoprotein traits, atherosclerosis, inflammation and pathogenic pathways during early arthritis stages. Rheumatology. 2023. doi:10.1093/rheumatology/kead009

19.	Szklarczyk D, Gable AL, Nastou KC, Lyon D, Kirsch R, Pyysalo S, et al. The STRING database in 2021: customizable protein–protein networks, and functional characterization of user-uploaded gene/measurement sets. Nucleic Acids Res. 2021;49:D605–12. doi:10.1093/nar/gkaa1074

20.	Zhou Y, Zhou B, Pache L, Chang M, Khodabakhshi AH, Tanaseichuk O, et al. Metascape provides a biologist-oriented resource for the analysis of systems-level datasets. Nat Commun. 2019;10:1523. doi:10.1038/s41467-019-09234-6

21.	Shannon P, Markiel A, Ozier O, Baliga NS, Wang JT, Ramage D, et al. Cytoscape: A Software Environment for Integrated Models of Biomolecular Interaction Networks. Genome Res. 2003;13:2498–504. doi:10.1101/gr.1239303

22.	Edgar R, Domrachev M, Lash AE. Gene Expression Omnibus: NCBI gene expression and hybridization array data repository. Nucleic Acids Res. 2002;30:207–10. PubMed PMID: 11752295.

**SUPPLEMENTARY TABLES**

**Supplementary Table 1: Demographic and clinical features of study participants.** The demographic and clinical features of the participants entered in this study were summarized. Variables were expressed as median (interquartile range) or n(%), unless otherwise stated, according to the distribution of the variables.

|  | **HC**  **n=65** | **CSA n=14** | **RA  n=82** |
| --- | --- | --- | --- |
| Age (years), mean (range) | 52.12  (36.10-71.20) | 49.28  (30.00-62.33) | 58.51  (30.33-83.67) |
| Gender (female/male) | 50/15 | 14/0 | 66/16 |
| ***Clinical features*** |  |  |  |
| Duration of symptoms (weeks) |  | 24.00 (40.00) | 20.00 (22.00) |
| Morning stiffness (minutes) |  | 30.00 (50.00) | 60.00 (80.0) |
| Tender joint count |  | 3.00 (3.00) | 8.00 (7.00) |
| Swollen joint count |  | 0.00 (1.00) | 6.00 (5.00) |
| Patient global assessment (VAS 0-100) |  | 30.00 (50.00) | 70.00 (25.00) |
| DAS28 |  |  | 5.40 (1.78) |
| DAS28 at 6 months (n=58) |  |  | 2.60 (1.94) |
| DAS28 at 12 months (n=58) |  |  | 2.19 (2.07) |
| HAQ |  | 0.55 (0.60) | 1.11 (1.00) |
| Pain (VAS 0-10) |  | 5.00 (5.00) | 7.00 (2.00) |
| RF+, n(%) |  | 8 (66.6) | 57 (69.5) |
| ACPA+, n(%) |  | 7 (58.3) | 56 (68.2) |
| ANA+, n(%) |  | 2 (14.2) | 34 (41.4) |
| ***Laboratory parameters*** |  |  |  |
| ESR (mm/h) |  | 7.50 (8.84) | 24.00 (27.00) |
| CRP (mg/dl) |  | 0.15 (0.30) | 0.80 (2.20) |
| Total cholesterol (mg/dl) |  | 196.50 (44.00) | 193.00 (48.00) |
| HDL-cholesterol (mg/dl) |  | 74.00 (15.59) | 50.00 (19.00) |
| LDL-cholesterol (mg/dl) |  | 116.00 (49.00) | 115.20 (47.00) |
| Triglycerides (mg/dl) |  | 74.50 (54.00) | 107.50 (80.00) |
| IgG (g/l) |  | 10.03 (3.33) | 10.99 (3.28) |
| IgA (g/l) |  | 1.97 (1.41) | 2.70 (1.38) |
| IgM (g/l) |  | 1.11 (0.73) | 1.11 (0.61) |
| C3 (g/l) |  | 1.23 (0.34) | 1.33 (0.42) |
| C4 (g/l) |  | 0.22 (0.08) | 0.29 (0.12) |
| ***Traditional CV risk factors, n(%)*** |  |  |  |
| Hypertension |  | 1 (12.5) | 28 (34.1) |
| Diabetes |  | 0 (0.0) | 9 (10.9) |
| Dyslipidemia |  | 3 (37.5) | 24 (29.2) |
| Smoking |  | 10 (71.4) | 31 (27.8) |
| Obesity (BMI>30 kg/m^2^) |  | 3 (37.5) | 33 (40.2) |
| ***Atherosclerosis burden*** |  | n=13 | n=77 |
| Plaque presence, n(%) |  | 4 (30.7) | 46 (59.7) |
| Plaque number, mean (range) |  | 0.46 (0-3) | 0.96 (0-4) |
| High-risk plaques, n(%) |  | 0 (0.0) | 20 (25.9) |
| cIMT (mm) |  | 0.58±0.15 | 0.67±0.10 |

**Supplementary Table 2: Demographic and parameters of established RA patients.** The demographic and clinical features of established RA patients recruited and matched HC as a validation cohort were shown. Variables were expressed as median (interquartile range), mean±SD or n(%), unless otherwise stated, according to the distribution of the variables.

|  | **HC**  **n=80** | **Established RA  n=103** |
| --- | --- | --- |
| Age (years), mean (range) | 51.05  (21.00 – 69.00) | 55.12  (22.00-87.00) |
| Gender (female/male) | 65/15 | 84/19 |
| ***Clinical features*** |  |  |
| Disease duration, median (range) (years) |  | 4.12 (1.00 – 21.13) |
| Age at diagnosis (years), mean (range) |  | 40.85 (18.00 – 80.92) |
| Tender joint count |  | 2.00 (6.50) |
| Swollen joint count |  | 1.00 (4.00) |
| Patient global assessment (VAS 0-100) |  | 30.00 (50.50) |
| Pain (VAS 0-10) |  | 3.00 (2.12) |
| DAS28 |  | 3.15 (2.25) |
| HAQ |  | 0.87 (1.14) |
| RF+, n(%) |  | 55 (53.3) |
| ACPA+, n(%) |  | 66 (64.0) |
| ANA+, n(%) |  | 46 (44.6) |
| History of CV events, n(%) |  | 16 (17.7) |
| ***Laboratory parameters*** |  |  |
| ESR (mm/h) |  | 15.00 (25.00) |
| CRP (mg/dl) |  | 1.00 (4.00) |
| Total cholesterol (mg/dl) |  | 203.00 (55.00) |
| HDL-cholesterol (mg/dl) |  | 62.00 (18.75) |
| LDL-cholesterol (mg/dl) |  | 118.00 (46.00) |
| Triglycerides (mg/dl) |  | 104.00 (73.25) |
| ***Traditional CV risk factors*** |  |  |
| Hypertension, n(%) |  | 38 (36.8) |
| Diabetes, n(%) |  | 8 (7.7) |
| Dyslipidemia, n(%) |  | 31 (30.1) |
| Smoking, n(%) |  | 30 (29.1) |
| Obesity (BMI>30 kg/m^2^), n(%) |  | 18 (17.4) |
| ***Treatments, n(%)*** |  |  |
| Glucocorticoids |  | 63 (61.1) |
| Methotrexate |  | 75 (72.8) |
| TNF blockers |  | 42 (40.7) |
| IL-6 blockers |  | 9 (8.7) |
| Leflunomide |  | 11 (10.6) |

**Supplementary Table 3: Demographic and parameters of SjD patients.** The demographic and clinical features of SjD patients recruited and matched HC were shown. Variables were expressed as median (interquartile range), mean±SD or n(%), unless otherwise stated, according to the distribution of the variables.

|  | **HC**  **n=45** | **SjD  n=44** |
| --- | --- | --- |
| Age (years), mean (range) | 63.51  (25.30-79.00) | 61.36  (35.00-80.00) |
| Gender (female/male) | 40/5 | 40/4 |
| ***Clinical features*** |  |  |
| Disease duration, median (range) (years) |  | 8.12 (5.10 – 25.01) |
| Xerostomia |  | 43 (97.7) |
| Xeroftalmia |  | 44 (100.0) |
| Extra-glandular manifestations |  | 31 (70.4) |
| ESSDAI |  | 5.11±5.08 |
| clinESSDAI |  | 4.45±4.74 |
| ANA+, n(%) |  | 39 (88.6) |
| Anti-RO/SSA+, n(%) |  | 38 (86.4) |
| Anti-LA/SSB+, n(%) |  | 16 (36.4) |
| RF+, n(%) |  | 25 (56.8) |
| ***Laboratory parameters*** |  |  |
| ESR (mm/h) |  | 20.00 (28.00) |
| CRP (mg/dl) |  | 1.00 (1.15) |
| Total cholesterol (mg/dl) |  | 100.50 (69.80) |
| HDL-cholesterol (mg/dl) |  | 60.50 (19.50) |
| LDL-cholesterol (mg/dl) |  | 117.00 (55.50) |
| Triglycerides (mg/dl) |  | 82.50 (60.00) |
| IgG (g/l) |  | 14.30 (8.02) |
| IgA (g/l) |  | 2.31 (1.31) |
| IgM (g/l) |  | 1.10 (0.74) |
| C3 (g/l) |  | 1.13 (0.38) |
| C4 (g/l) |  | 0.20 (0.14) |
| ***Traditional CV risk factors*** |  |  |
| Hypertension, n(%) |  | 13 (29.5) |
| Diabetes, n(%) |  | 5 (11.4) |
| Dyslipidemia, n(%) |  | 6 (13.6) |
| Smoking, n(%) |  | 8 (18.2) |
| Obesity (BMI>30 kg/m^2^), n(%) |  | 10 (22.7) |
| ***Treatments, n(%)*** |  |  |
| Hydroxychloroquine |  | 23 (52.3) |
| Glucocorticoids |  | 14 (31.8) |
| Methotrexate |  | 4 (9.1) |
| Azathioprine |  | 3 (6.8) |
| Rituximab |  | 3 (6.8) |
| ***Atherosclerosis burden*** |  |  |
| Plaque presence, n(%) |  | 22 (50.0) |
| Plaque number, mean (range) |  | 0.67 (0-4) |
| cIMT (mm) |  | 0.62±0.10 |

**Supplementary Table 4: Associations between anti-CXCR3 levels and cytokines.** Associations between serum levels of anti-CXCR3 antibodies and cytokines in early RA were analysed by Spearman’s rank tests in early RA. Coefficients (r) and p-values are shown. Those reaching statistical significance were highlighted in bold.

|  | **Anti-CXCR3** |
| --- | --- |
| IL-6 | r=-0.097  p=0.388 |
| TNF | r=-0.162  p=0.145 |
| IFNg | r=-0.147  p=0.187 |
| IL-1b | r=-0.068  p=0.545 |
| IL-23 | r=-0.092  p=0.413 |
| IL-12 | r=-0.123  p=0.269 |
| IL-33 | r=-0.079  p=0.478 |
| IL-10 | r=-0.082  p=0.461 |
| IL-17 | r=0.169  p=0.130 |
| IL-8 | r=-0.008  p=0.942 |
| IL-18 | r=0.024  p=0.828 |
| IL-21 | r=-0.080  p=0.550 |
| APRIL | r=-0.046  p=0.733 |
| BAFF | r=-0.061  p=0.648 |

**Supplementary Table 5: Demographic and clinical features of RA patients undergoing TNFi treatment.** The demographic, clinical features and treatment outcomes at 3 months of biological-naïve RA patients undergoing TNFi were summarized. Variables were expressed as median (interquartile range), mean±SD or n(%), unless otherwise stated.

|  | **Biological-naïve RA n=13** | |
| --- | --- | --- |
| Age (years), mean (range) | 46.71  (30.75-65.42) | |
| Gender (female/male) | 12/1 | |
| ***Clinical features*** |  |  |
| Disease duration, median (range) (years) | 1.54 (1.00 – 7.17) | |
| Age at diagnosis (years), mean (range) | 44.28 (29.08 – 62.75) | |
| RF+, n(%) | 5 (38.4) | |
| ACPA+, n(%) | 6 (46.1) | |
| ANA+, n(%) | 5 (38.4) | |
| ***Treatment outcomes*** | *Baseline* | *3 months* |
| Tender joint count | 9.00 (5.00) | 4.50 (3.00) |
| Swollen joint count | 5.00 (3.00) | 3.00 (1.69) |
| ESR (mm/h) | 13.00 (28.00) | 9.5 (16.25) |
| CRP (mg/dl) | 3.00 (4.15) | 1.00 (1.50) |
| Patient global assessment (VAS 0-100) | 66.00 (17.00) | 35.00 (20.28) |
| Pain (VAS 0-10) | 6.00 (2.90) | 3.25 (2.97) |
| DAS28 | 5.15 (1.99) | 3.80 (1.97) |
| HAQ | 1.20 (0.79) | 0.87 (0.85) |

**Supplementary Table 6: Anti-CXCR3 antibodies as predictors of atherosclerosis in established RA.** The role of anti-CXCR3 levels as predictor of atherosclerosis occurrence in established RA patients (validation cohort) was analysed by univariate and multivariate logistic regression models (including CV risk factors and disease duration). Odds ratio (OR), 95% confidence intervals (95% CI) and p-values for each predictor were shown. Those reaching statistical significance were highlighted in bold.

|  | **OR** | **95% CI** | **p-value** |
| --- | --- | --- | --- |
| ***Univariate*** | | | |
| Anti-CXCR3, per unit | 0.972 | 0.910 – 0.994 | **0.030** |
| ***Multivariate*** | | | |
| Anti-CXCR3, per unit | 0.970 | 0.850 – 0.996 | **0.029** |
| Sex, women | 0.333 | 0.061 – 1.827 | 0.206 |
| Age, per year | 1.100 | 1.011 – 1.197 | **0.026** |
| Smoking, yes | 1.562 | 0.272 – 8.975 | 0.617 |
| Hypertension, yes | 3.712 | 0.835 – 16.492 | 0.085 |
| Diabetes, yes | 2.714 | 0.280 – 6.258 | 0.398 |
| Dyslipidemia, yes | 0.891 | 0.190 – 4.225 | 0.891 |
| Disease duration, per year | 1.073 | 0.952 – 1.209 | 0.617 |

**Supplementary Table 7: Anti-CXCR3 antibodies as predictors of atherosclerosis in established RA.** The role of anti-CXCR3 levels as predictor of atherosclerosis occurrence in established RA patients (validation cohort) was analysed by univariate and multivariate logistic regression models (including CV risk factors and disease duration, and treatment exposure). IL-6 blockers and leflunomide were not entered in the analysis due to reduced sample size (<10% of patients). Odds ratio (OR), 95% confidence intervals (95% CI) and p-values for each predictor were shown. Those reaching statistical significance were highlighted in bold.

|  | **OR** | **95% CI** | **p-value** |
| --- | --- | --- | --- |
| ***Univariate*** | | | |
| Anti-CXCR3, per unit | 0.972 | 0.910 – 0.994 | **0.030** |
| ***Multivariate*** | | | |
| Anti-CXCR3, per unit | 0.965 | 0.832 – 0.995 | **0.027** |
| Sex, women | 0.275 | 0.038 – 1.987 | 0.201 |
| Age, per year | 1.110 | 1.017 – 1.211 | **0.020** |
| Smoking, yes | 1.755 | 0.125 – 5.052 | 0.736 |
| Hypertension, yes | 4.156 | 0.981 – 16.102 | 0.053 |
| Diabetes, yes | 3.372 | 0.263 – 7.152 | 0.350 |
| Dyslipidemia, yes | 1.001 | 0.188 – 5.322 | 0.999 |
| Disease duration, per year | 1.034 | 0.894 – 1.197 | 0.649 |
| Glucocorticoids, yes | 3.431 | 0.646 – 9.231 | 0.348 |
| Methotrexate, yes | 3.989 | 0.660 – 6.241 | 0.150 |
| TNF blockers, yes | 0.620 | 0.106 – 3.619 | 0.596 |

**Supplementary Table 8: Anti-CXCR3 antibodies as predictors of atherosclerosis in SjD.** The role of anti-CXCR3 levels as predictor of atherosclerosis occurrence in SjD patients was analysed by univariate and multivariate logistic regression models. Sex was omitted from the multivariate analysis due to sample size limitations. Odds ratio (OR), 95% confidence intervals (95% CI) and p-values for each predictor were shown. Those reaching statistical significance were highlighted in bold.

|  | **OR** | **95% CI** | **p-value** |
| --- | --- | --- | --- |
| ***Univariate*** | | | |
| Anti-CXCR3, per unit | 0.826 | 0.684 – 0.996 | **0.046** |
| ***Multivariate*** | | | |
| Anti-CXCR3, per unit | 0.641 | 0.421 – 0.912 | **0.039** |
| Age, per year | 1.291 | 1.079 – 1.540 | **0.005** |
| Smoking, yes | 0.505 | 0.096 – 2.660 | 0.421 |
| Hypertension, yes | 0.939 | 0.127 – 6.947 | 0.951 |
| Diabetes, yes | 0.502 | 0.001 – 6.566 | 0.378 |
| Dyslipidemia, yes | 1.378 | 0.005 – 16.588 | 0.919 |
| Disease duration, per year | 0.981 | 0.840 – 1.145 | 0.804 |

**Supplementary Table 9: Anti-CXCR3 antibodies as predictors of atherosclerosis in SjD.** The role of anti-CXCR3 levels as predictor of atherosclerosis occurrence in SjD patients was analysed by univariate and multivariate logistic regression models (including traditional CV risk factors, disease duration and treatment exposures). Sex was omitted from the multivariate analysis due to sample size limitations. Similarly, methotrexate, azathioprine and rituximab were not entered in the analysis due to reduced sample size (<10% of patients). Odds ratio (OR), 95% confidence intervals (95% CI) and p-values for each predictor were shown. Those reaching statistical significance were highlighted in bold.

|  | **OR** | **95% CI** | **p-value** |
| --- | --- | --- | --- |
| ***Univariate*** | | | |
| Anti-CXCR3, per unit | 0.826 | 0.684 – 0.996 | **0.046** |
| ***Multivariate*** | | | |
| Anti-CXCR3, per unit | 0.689 | 0.479 – 0.989 | **0.041** |
| Age, per year | 1.295 | 1.077 – 1.558 | **0.006** |
| Smoking, yes | 0.510 | 0.094 – 2.775 | 0.436 |
| Hypertension, yes | 1.027 | 0.135 – 7.819 | 0.981 |
| Diabetes, yes | 0.902 | 0.032 – 8.379 | 0.674 |
| Dyslipidemia, yes | 1.413 | 0.007 – 7.819 | 0.981 |
| Disease duration, per year | 0.915 | 0.728 – 1.156 | 0.963 |
| Hydroxychloroquine, yes | 0.651 | 0.065 – 5.717 | 0.878 |
| Glucocorticoids, yes | 0.862 | 0.130 – 1.156 | 0.686 |
